# Supplementary material for: Slow Adaptive Response of Budding Yeast Cells to Stable Conditions of Continuous Culture Can Occur without Genome Modifications
Source: Genes (Basel). 2020 Nov 27;11(12):1419. doi: 10.3390/genes11121419 (PMC7759791; doi:10.3390/genes11121419)
Supplement: Supplementary file 1 [file genes-11-01419-s001.zip › Supplementary data revised.pdf]

Supplementary data

for

Slow adaptive response of budding yeast cells to stable conditions of continuous culture can occur without genome modifications

Joanna Klim [1], Urszula Zielenkiewicz [2], Anna Kurlandzka [3] , Szymon Kaczanowski [4] and Marek Skoneczny [5,*]

Description of the microevolution study as the source of data described in this manuscript

The data analyzed in this manuscript are derived from a larger microevolution study testing the hypothesis that compensatory evolutionary modifications, such as mutations and transcriptional changes, occur frequently in genes from perturbed modules of genetically interacting genes. The modules consist of functionally related genes encoding proteins that may form complexes and/or participate in common biochemical pathways. The deletions of two genes encoding evolutionarily conserved *Saccharomyces cerevisiae* proteins: Cog7p and Nup133p likely created perturbations in two such modules. In the laboratory microevolution experiments (carried out for more than 200 generations) we were following the accumulation of compensatory genome and transcriptome changes in yeast cell populations lacking the respective *COG7* or *NUP133* genes. Parental WT strain (W303 genetical background), subject to the same experimental conditions was used as a reference. Each strain was subjected to microevolution in three independent biological replicates. To accelerate evolution a separate set of continuous cultures was performed with *cog7Δ*, *nup133Δ* and a reference strain, all carrying an additional deletion of *MSH1* gene, which conferred the mutator phenotype.

When establishing continuous culture conditions for this study, our intention was to avoid any selection pressure, which would otherwise enforce the gradual accumulation and enrichment of adaptive mutations in the cultured cell populations and bias our results towards adaptation to that pressure. To this end, we did not apply nutrient limitation, which would cause starvation stress. The steadiness of population growth was assured by gauging the medium flow rate to the growth rate rather than enforcing the population doubling time by the flow rate of nutrient-limited growth medium, as is common in chemostat culture. A maintained density of 5.3 ±0.3 x 10^7 cells per mL, equivalent to the late exponential phase of batch culture, assured constant, unperturbed growth of the cells. Moreover, the selection of synthetic minimal medium with glycerol as a respiratory carbon source increased the demand for the cells' metabolic capacity, hence maximizing the number of genes that were expressed in them.

The evolved yeast cell populations were characterized through whole genome sequencing and whole transcriptome analyses. Numerous mutations in the genomes and numerous changes in the transcriptome were identified, especially in cell populations lacking *MSH1* gene. The extensive analysis of these data is a subject of another manuscript.

Results of microevolution experiment for the wild-type reference strain

As one could expect the wild-type, reference W303 cell populations accumulated the fewest significant mutations, i.e. leading to modification of proteins. The list of them is given below:

Replicate 1 (Rep-1)

Short deletion in *HOG1* gene leading to frameshift. Mutated gene encoded truncated 243 aa protein, most likely inactive.

A mutation in *MSY1* gene leading to conservative Leu287→Phe substitution in the encoded protein.

Replicate 2 (Rep-2)

A point mutation in *HOG1* gene leading to Glu186→Lys substitution in the encoded protein.

A point mutation in *URE2* gene leading to Gln209→STOP and truncation of Ure2p.

Replicate 3 (Rep-3)

A mutation in *MSY1* gene leading to conservative Leu287→Phe substitution in the encoded protein.

HOG1 gene encodes mitogen-activated protein kinase involved in osmoregulation, *URE2* gene encodes nitrogen catabolite repression transcriptional regulator and *MSY1* gene encodes mitochondrial tyrosyl-tRNA synthetase.

The presence of these mutations, especially in *HOG1* and *URE2* genes encoding proteins involved in cellular signaling and regulation of cellular processes indicates that WT W303 strain was subject to some evolutionary drift as well. In fact, the mutations inactivating regulatory proteins were previously reported and the loss of signaling networks was suggested as an adaptation to continuous culture conditions in other microevolution studies [1]. Since there were no genes that were mutated in all biological replicates one could expect the differences in the expression of genes between individual biological replicates; indeed such differences were present. When comparing Log_2Ratio data for genes that responded differently to continuous culture conditions in three biological replicates (see Table S3) we noticed that Rep-1 was the one that diverged the most from the remaining two biological replicates. The difference between the data for Rep-1 and the average of Rep-2 and Rep-3 biological replicates was above 0.9 or below -0.9 for 416 genes. The differences between Rep-2 vs the average of Rep-1 and Rep-3 and between Rep-3 vs the average of Rep-1 and Rep-2 were above 0.9 or below -0.9 for 79 and 171 genes, respectively. Much weaker presumable effect of *HOG1* mutation in Rep-2 may be explained by the fact that the *HOG1* mutation in Rep-1 was a short deletion with frameshift leading to truncated Hog1p and its inactivation, whereas in Rep-2 there was a point mutation in *HOG1*, likely with the minor effect on the function of encoded protein and the expression of controlled genes. Regarding *MSY1* and *URE2* we cannot distinguish the effect of mutations in these genes, because the mutation in *URE2* was detected in Rep-2 cell population, whereas the mutation in *MSY1* was detected in Rep-1 and Rep-3 cell populations. In *MSY1* gene we detected a point mutation leading to conservative Leu→Phe substitution whereas in *URE2* we identified much more significant mutation, a STOP introduced after 208 codon

leading to truncation and probable inactivation of encoded protein. Both these mutations, as revealed by calculating the difference in the Log_2Ratio between the data for Rep-2 and the average of the other two biological replicates, affected the expression of 79 genes. The difference between the data for Rep-3 and the average of Rep-1 and Rep-2 observed for 171 genes probably reflects the combined effect of mutations in *HOG1* and *URE2* genes.

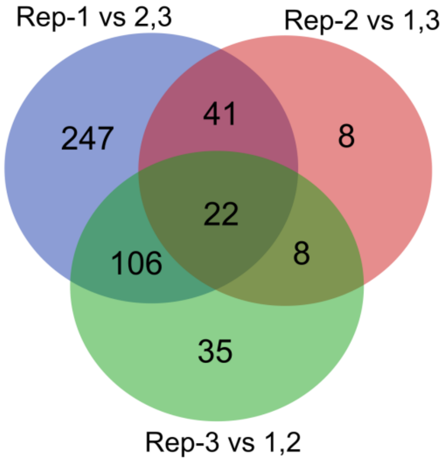

Figure S1. Venn diagram showing the number of genes whose expression under continuous culture conditions was commonly or differently affected by mutations in *MSY1, HOG1* and *URE2* genes. See Table S3 for more information on individual genes.

As demonstrated by the Venn diagram in Figure S1 these gene sets are overlapping, which is not surprising because in all three biological replicates the effects of single or combined inactivation of *MSY1, HOG1* and *URE2* genes were exposed. The total number of genes that met at least one of the criteria mentioned above was 467.

One could treat these calculations with reluctance, arguing that they are based on single biological replicates, therefore are not statistically significant and could be the result of experimental imperfections rather than mutations found in the genomes of cell populations. However, and this is the phenomenon we describe and analyze in the main text of this paper, there were more than four hundred genes whose change in expression between 40th and 240th generations was uniform in all biological replicates. This result proves the integrity of transcriptome data and ensures that the differences observed between the biological replicates for some genes are genuine and reflect the possible consequence of Hog1p or Ure2p inactivation.

Reference

1. Kvitek, D.J.; Sherlock, G. Whole genome, whole population sequencing reveals that loss of signaling networks is the major adaptive strategy in a constant environment. *PLoS Genet*. **2013**, 9, e1003972, doi:10.1371/journal.pgen.1003972.

Table S1 Primers used for verification of the transcriptome data with qRT-PCR

**Primer name**	**Targeted PCR product**	**Primer sequence (5'→3')**
ACT1F	*ACT1* (reference)	AGAGTTGCCCCAGAAGAACA
ACT1R		GGCTTGGATGGAAACGTAGA
L_YLR413W_1150-1449	*INA1*	TGACAACTATCACACCAGAACA
R_YLR413W_1150-1449		TTGTTCTGTTTAAGTGAGCACG
L_YJR004C_350-649	*SAG1*	GATGTGGTGAATTTCGATCCTG
R_YJR004C_350-649		TCAATCTTCTCAGTACCACCC
L_YKR026C_0-299	*GCN3*	ATACTGAGATGACAATGCCGAT
R_YKR026C_0-299		GAAAATATCACAACCGGCTCTC
L_YHR196W_50-349	*UTP9*	CTTTGGGTGGTGTAGTGATTTG
R_YHR196W_50-349		AAACAACAATTCTTCCGTCAGG
L_YOL104C_400-699	*NDJ1*	CCGTTTTAACAAAATTTGGCCC
R_YOL104C_400-699		CATTCGGAATGTTCTGATGGAC
L_YER126C_400-699	*NSA2*	CACTCATCCGGAATTAGGTGTA
R_YER126C_400-699		CCCAATTCCGAAACGTTAACTT
L_YHR065C_600-899	*RRP3*	GACATGGAATTTGGACCTGTTC
R_YHR065C_600-899		ACAGGATTTGTCAAACTTGCTC
L_YHR088W_50-349	*RPF1*	CACTAGAGTGTACGATGAGACC
R_YHR088W_50-349		TTTGGTGGCTCATTAGAATTGC

Figure S2 Correlation between the transcriptome and qRT-PCR data. The calculated correlation coefficient was 0.931. See Table S1 above for the list of genes selected for this experiment.

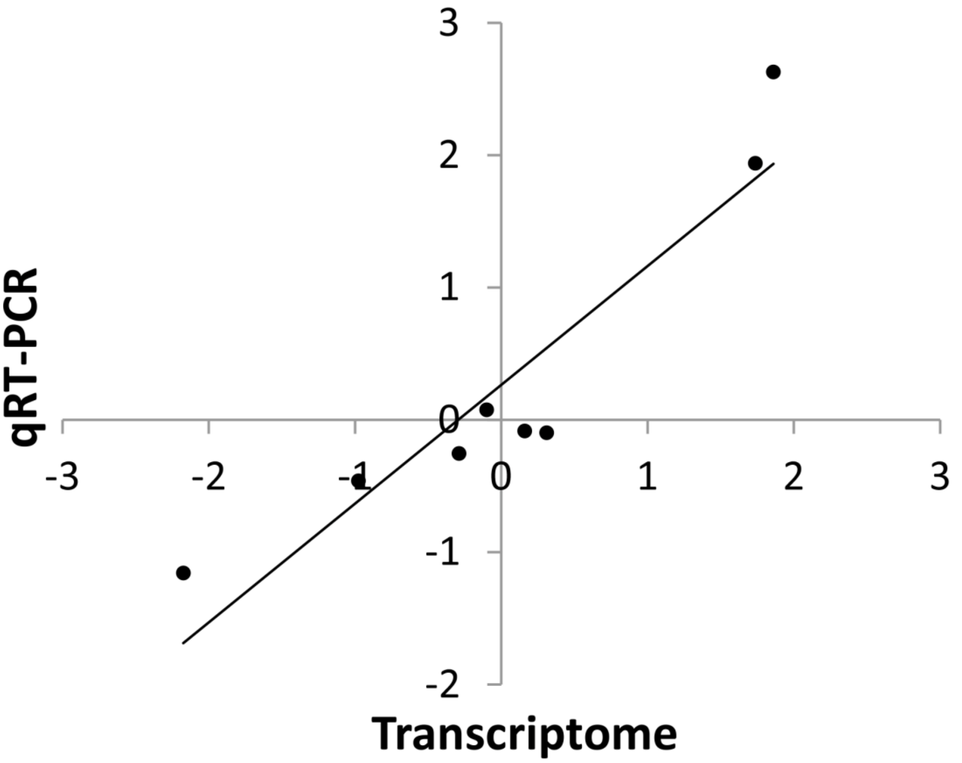

Figure S3 All upregulated and downregulated genes selected in the course of transcriptome analysis sorted according to functional category assigned to them. For clarity, the number of GO terms, shown in Figure 1, was reduced by merging the lists of genes of similar functions. Sorting was done manually on the basis of GO annotations assigned to genes and the description of their function in Saccharomyces Genome Database (SGD). Below the gray bar are genes whose functions were not overrepresented within the gene-sets. Leftmost column shows the Log2Ratio of change in gene expression between 40th and 240th generation of growth in continuous culture. Rightmost column displays short description of gene function taken from SGD.

Up-regulated genes

Log2Ratio color scale

Log2Ratio
-4
-3
-2
-1
0
1
2
3
4

Peroxisome

Log2Ratio	ORF	Gene	Description
1.144	YDR256C	CTA1	Catalase A
1.999	YIR031C	DAL7	Malate synthase
1.170	YLR284C	ECI1	Peroxisomal delta3,delta2-enoyl-CoA isomerase
1.215	YER015W	FAA2	Medium chain fatty acyl-CoA synthetase
1.709	YKR009C	FOX2	3-OH-acyl-CoA dehydrogenase / enoyl-CoA hydratase
1.388	YNL009W	IDP3	Peroxisomal isocitrate dehydrogenase
1.379	YOR084W	LPX1	Peroxisomal matrix-localized lipase
2.207	YGL205W	POX1	Fatty-acyl coenzyme A oxidase
1.045	YNL202W	SPS19	Peroxisomal 2,4-dienoyl-CoA reductase

Catabolism of small molecules, including aminoacids

Log2Ratio	ORF	Gene	Description
1.904	YMR303C	ADH2	Glucose-repressible alcohol dehydrogenase II
0.914	YMR170C	ALD2	Cytoplasmic aldehyde dehydrogenase
1.878	YLR155C	ASP3-1	Cell-wall L-asparaginase II - asparagine catabolism
1.859	YLR157C	ASP3-2	Cell-wall L-asparaginase II - asparagine catabolism
1.894	YLR158C	ASP3-3	Cell-wall L-asparaginase II - asparagine catabolism
1.867	YLR160C	ASP3-4	Cell-wall L-asparaginase II - asparagine catabolism
1.528	YJR148W	BAT2	Cytosolic branched-chain amino acid aminotransferase
0.942	YOR125C	CAT5	Protein required for ubiquinone (CoQ) biosynthesis
2.357	YLR307W	CDA1	Chitin deacetylase
1.320	Q0250	COX2	Subunit II of cytochrome c oxidase (Complex IV)
2.932	YIR027C	DAL1	Allantoinase
3.249	YIR028W	DAL4	Allantoin permease
4.073	YJR152W	DAL5	Allantoate permease
2.403	YHL016C	DUR3	Plasma membrane urea and polyamines transporter
0.976	YML080W	DUS1	Dihydrouridine synthase
1.157	YOR384W	FRE5	Putative ferric reductase with similarity to Fre2p
1.171	YDL215C	GDH2	NAD(+)-dependent glutamate dehydrogenase
1.147	YBR248C	HIS7	Imidazole glycerol phosphate synthase
1.431	YEL066W	HPA3	D-Amino acid N-acetyltransferase
0.955	YNL239W	LAP3	Cysteine aminopeptidase
1.134	YCL018W	LEU2	Beta-isopropylmalate dehydrogenase (IMDH)
1.349	YBR213W	MET8	Bifunctional dehydrogenase and ferrochelatase
1.829	YPL171C	OYE3	Conserved NADPH oxidoreductase
1.152	YER023W	PRO3	Delta 1-pyrroline-5-carboxylate reductase
1.440	YKL132C	RMA1	Putative dihydrofolate synthetase
0.995	YIL167W	SDL1	Blocked reading frame encoding L-serine dehydratase
1.338	YAL067C	SEO1	Putative permease
1.132	YPR069C	SPE3	Spermidine synthase
1.302	YJR103W	URA8	Minor CTP synthase isozyme (see also URA7)

Mitochondrion

1.105	YOR374W	ALD4	Mitochondrial aldehyde dehydrogenase
2.117	YER073W	ALD5	Mitochondrial aldehyde dehydrogenase
1.120	YNL315C	ATP11	Molecular chaperone
1.387	YIL098C	FMC1	Mitochondrial matrix protein
1.214	YPR006C	ICL2	2-methylisocitrate lyase of the mitochondrial matrix
0.930	YCR071C	IMG2	Mitochondrial ribosomal protein of the large subunit
0.953	YIL070C	MAM33	Translational activator for mitochondrial COX1 mRNA
0.984	YDR296W	MHR1	Mitochondrial ribosomal protein of the large subunit
1.196	YGR084C	MRP13	Mitochondrial ribosomal protein of the small subunit
1.149	YOR150W	MRPL23	Mitochondrial ribosomal protein of the large subunit
1.040	YMR024W	MRPL3	Mitochondrial ribosomal protein of the large subunit
0.968	YKL138C	MRPL31	Mitochondrial ribosomal protein of the large subunit
1.422	YMR225C	MRPL44	Mitochondrial ribosomal protein of the large subunit
1.016	YMR228W	MTF1	Mitochondrial RNA polymerase specificity factor
2.409	YLR142W	PUT1	Proline oxidase
0.938	YNL122C	YNL122C	Mitochondrial ribosomal protein of the large subunit

Sister chromatid segregation

1.335	YMR198W	CIK1	Kinesin-associated protein
1.137	YMR048W	CSM3	Replication fork associated factor
1.202	YPL209C	IPL1	Aurora kinase of chromosomal passenger complex
1.019	YIL026C	IRR1	Subunit of the cohesin complex
1.351	YDL003W	MCD1	Essential alpha-kleisin subunit of the cohesin complex
1.317	YDR113C	PDS1	Securin
1.015	YOR073W	SGO1	Component of the spindle checkpoint
0.944	YFL008W	SMC1	Subunit of the multiprotein cohesin complex
1.097	YJL074C	SMC3	Subunit of the multiprotein cohesin complex
1.151	YLR212C	TUB4	Gamma-tubulin

Purine nucleotide salvage

1.551	YDR399W	HPT1	Hypoxanthine-guanine phosphoribosyltransferase
1.539	YJR133W	XPT1	Xanthine-guanine phosphoribosyl transferase

Signal transduction

0.967	YDR277C	MTH1	Negative regulator of the glucose-sensing signal transduction pathway
2.691	YOL128C	YGK3	Protein kinase related to mammalian GSK-3 glycogen synthase kinases

Response to toxin

1.729	YFL061W	DDI2	Cyanamide hydratase that detoxifies cyanamide
1.713	YNL335W	DDI3	Cyanamide hydratase that detoxifies cyanamide

Cell division

1.113	YLR353W	BUD8	Protein involved in bud-site selection
0.950	YHR107C	CDC12	Component of the septin ring that is required for cytokinesis
1.075	YDL161W	ENT1	Epsin-like protein involved in endocytosis and actin patch assembly
1.012	YER032W	FIR1	Protein involved in 3' mRNA processing
1.202	YDR507C	GIN4	Protein kinase involved in bud growth and assembly of the septin ring
1.033	YOR181W	LAS17	Actin assembly factor
1.375	YLR313C	SPH1	Protein involved in shmoo formation and bipolar bud site selection
1.458	YGR221C	TOS2	Protein involved in localization of Cdc24p to the site of bud growth
1.079	YML109W	ZDS2	Protein with a role in regulating Swe1p-dependent polarized growth

Metabolism

1.510	YOR074C	CDC21	Thymidylate synthase
1.776	YNL129W	NRK1	Nicotinamide riboside kinase
1.195	YOR143C	THI80	Thiamine pyrophosphokinase
1.204	YPL201C	YIG1	Protein that interacts with glycerol 3-phosphatase

Protein modificatiopn

1.613	YPR051W	MAK3	Catalytic subunit of the NatC type N-terminal acetyltransferase (NAT)
0.919	YOR281C	PLP2	Protein that interacts with the CCT complex to stimulate actin folding
1.015	YJR099W	YUH1	Ubiquitin C-terminal hydrolase

Endomembrane system

1.270	YGR142W	BTN2	v-SNARE binding protein
0.963	YLR246W	ERF2	Subunit of a palmitoyltransferase
1.337	YML008C	ERG6	Delta(24)-sterol C-methyltransferase
0.999	YML104C	MDM1	PtdIns-3-P binding protein that tethers the ER to vacuoles at NVJs
0.954	YHL023C	NPR3	Subunit of the Iml1p/SEACIT complex
6.586	YPR194C	OPT2	Oligopeptide transporter
1.160	YGL226C-A	OST5	Zeta subunit of the oligosaccharyltransferase complex of the ER lumen
1.012	YOR216C	RUD3	Golgi matrix protein
0.941	YJL145W	SFH5	Non-classical phosphatidylinositol transfer protein (PITP)
0.900	YBR264C	YPT10	Rab family GTP-binding protein
1.191	YER031C	YPT31	Rab family GTPase
1.057	YOR003W	YSP3	Putative precursor of the subtilisin-like protease III

Extracellular region

3.158	YGL089C	MF(ALPHA)2	Mating pheromone alpha-factor, made by alpha cells
2.049	YJR153W	PGU1	Endo-polygalacturonase

Mitochondrial metabolism

1.181	YBR085W	AAC3	Mitochondrial inner membrane ADP/ATP translocator
2.307	YNR020C	ATP23	Putative metalloprotease of the mitochondrial inner membrane
0.938	YOL096C	COQ3	O-methyltransferase
1.237	YLL009C	COX17	Copper metallochaperone that transfers copper to Sco1p and Cox11p
1.000	YHR116W	COX23	Protein that functions in mitochondrial copper homeostasis
1.056	YLR348C	DIC1	Mitochondrial dicarboxylate carrier
0.996	YIL134W	FLX1	Mitochondrial flavin adenine dinucleotide transporter
0.979	YOL023W	IFM1	Mitochondrial translation initiation factor 2
1.256	YKL120W	OAC1	Mitochondrial inner membrane transporter
0.993	YML030W	RCF1	Cytochrome c oxidase subunit
1.229	YAR008W	SEN34	Subunit of the tRNA splicing endonuclease
1.073	YJR135W-A	TIM8	Mitochondrial intermembrane space protein
1.419	YBR104W	YMC2	Putative mitochondrial inner membrane transporter

Chromatin-associated

0.995	YIR010W	DSN1	Essential component of the MIND kinetochore complex
1.106	YNL164C	IBD2	Component of the BUB2-dependent spindle checkpoint pathway
1.018	YKL089W	MIF2	Protein required for structural integrity of elongating spindles
1.058	YPR018W	RLF2	Largest subunit (p90) of the Chromatin Assembly Complex (CAF-1)
0.955	YFR037C	RSC8	Component of the RSC chromatin remodeling complex
1.012	YHR207C	SET5	Methyltransferase involved in methylation of histone H4 Lys5, -8, -12
1.077	YHR127W	YHR127W	Protein of unknown function

DNA repair

0.900	YGL090W	LIF1	Component of the DNA ligase IV complex
1.075	YML060W	OGG1	Nuclear and mitochondrial glycosylase/lyase
1.159	YJR043C	POL32	Third subunit of DNA polymerase delta
1.012	YKL113C	RAD27	5' to 3' exonuclease, 5' flap endonuclease

Transcription factors

1.594	YKR034W	DAL80	Negative regulator of genes in multiple nitrogen degradation pathways
0.996	YIL131C	FKH1	Forkhead family transcription factor
1.341	YMR136W	GAT2	Protein containing GATA family zinc finger motifs
1.559	YDR123C	INO2	Transcription factor
3.152	YGL209W	MIG2	Zinc finger transcriptional repressor
1.164	YER028C	MIG3	Transcriptional regulator
1.591	YNL289W	PCL1	Cyclin, interacts with cyclin-dependent kinase Pho85p
1.856	YOR315W	SFG1	Nuclear protein putative transcription factor
0.966	YPR009W	SUT2	Zn2Cys6 family transcription factor
1.077	YBR083W	TEC1	Transcription factor targeting filamentation genes and Ty1 expression
1.324	YLR183C	TOS4	Putative transcription factor, contains Forkhead Associated domain
1.343	YDR451C	YHP1	Homeobox transcriptional repressor
1.329	YHR177W	YHR177W	Putative transcription factor containing a WOPR domain

RNA metabolism

1.027	YGR246C	BRF1	TFIIIB B-related factor
0.972	YKL139W	CTK1	Catalytic (alpha) subunit of C-terminal domain kinase I (CTDK-I)
1.034	YHR169W	DBP8	ATPase, putative RNA helicase of the DEAD-box family
1.203	YHR085W	IPI1	Component of the Rix1 complex and possibly pre-replicative complexes
1.229	YOR274W	MOD5	Delta 2-isopentenyl pyrophosphate:tRNA isopentenyl transferase
0.933	YDR243C	PRP28	RNA binding protein
0.940	YPL208W	RKM1	SET-domain lysine-N-methyltransferase
1.136	YML043C	RRN11	Component of the core factor (CF) rDNA transcription factor complex
1.629	YIL082W	YIL082W	Retrotransposon TYA Gag gene co-transcribed with TYB Pol

Plasma membrane transport and signaling

1.136	YML006C	GIS4	CAAX box containing protein of unknown function
0.971	YNR072W	HXT17	Putative transmembrane polyol transporter
1.241	YDR343C	HXT6	High-affinity glucose transporter
1.262	YIL120W	QDR1	Multidrug transporter of the major facilitator superfamily
1.063	YBR043C	QDR3	Multidrug transporter of the major facilitator superfamily
0.967	YER118C	SHO1	Transmembrane osmosensor for filamentous growth and HOG pathways

Unknown function

1.462	YMR003W	AIM34	Protein of unknown function
1.888	YNL269W	BSC4	Protein of unknown function
1.721	YIR030C	DCG1	Protein of unknown function
1.090	YOR271C	FSF1	Putative protein
1.447	YOL056W	GPM3	Homolog of Gpm1p phosphoglycerate mutase
1.085	YNL215W	IES2	Protein that associates with the INO80 chromatin remodeling complex
1.202	YDR316W	OMS1	Protein integral to the mitochondrial membrane
1.008	YLR338W	OPI9	Dubious open reading frame
1.458	YDR281C	PHM6	Protein of unknown function
1.720	Q0010	Q0010	Dubious open reading frame
1.364	Q0182	Q0182	Dubious open reading frame
1.162	Q0297	Q0297	Dubious open reading frame
1.289	YDL048C	STP4	Protein containing a Kruppel-type zinc-finger domain

1.589	YPR157W	TDA6	Putative protein of unknown function
1.735	YAR061W	YAR061W	Pseudogenic fragment with similarity to flocculins
0.916	YBL010C	YBL010C	Putative protein of unknown function
2.121	YBL065W	YBL065W	Dubious open reading frame
1.045	YBL107W-A	YBL107W-A	Dubious open reading frame
1.006	YBR206W	YBR206W	Dubious open reading frame
2.105	YCL021W-A	YCL021W-A	Putative protein of unknown function
1.657	YDL114W	YDL114W	Putative short-chain dehydrogenase/reductase
1.772	YDL211C	YDL211C	Protein of unknown function
1.676	YEL076C	YEL076C	Putative protein of unknown function
1.808	YEL076C-A	YEL076C-A	Putative protein of unknown function
1.142	YER138W-A	YER138W-A	Putative protein of unknown function
1.173	YFL064C	YFL064C	Putative protein of unknown function
1.419	YFL065C	YFL065C	Putative protein of unknown function
1.465	YFL067W	YFL067W	Protein of unknown function
0.937	YFL068W	YFL068W	Putative protein of unknown function
1.247	YGL242C	YGL242C	Protein of unknown function
1.055	YGR021W	YGR021W	Putative protein of unknown function
2.545	YGR190C	YGR190C	Dubious open reading frame
1.143	YHL026C	YHL026C	Putative protein of unknown function
1.413	YHL049C	YHL049C	Putative protein of unknown function
1.584	YHR213W	YHR213W	Pseudogenic fragment with similarity to flocculins
1.041	YHR214W-A	YHR214W-A	Dubious open reading frame
1.361	YHR217C	YHR217C	Dubious open reading frame
1.479	YIL174W	YIL174W	Hypothetical protein
1.619	YIR040C	YIR040C	Dubious open reading frame
0.970	YJL055W	YJL055W	Putative protein of unknown function
1.217	YJR011C	YJR011C	Putative protein of unknown function
1.246	YKR075C	YKR075C	Protein of unknown function
1.117	YLR257W	YLR257W	Protein of unknown function
1.051	YLR379W	YLR379W	Dubious open reading frame
1.892	YLR464W	YLR464W	Putative protein of unknown function
1.107	YML034C-A	YML034C-A	Dubious open reading frame
1.688	YML122C	YML122C	Putative protein of unknown function
1.334	YMR007W	YMR007W	Putative protein of unknown function
1.030	YMR082C	YMR082C	Putative protein of unknown function
1.167	YMR193C-A	YMR193C-A	Dubious open reading frame
0.907	YNL043C	YNL043C	Dubious open reading frame
1.134	YNR029C	YNR029C	Putative protein of unknown function
1.054	YOR200W	YOR200W	Dubious open reading frame
1.101	YOR263C	YOR263C	Dubious open reading frame
1.168	YOR331C	YOR331C	Dubious open reading frame
1.175	YOR389W	YOR389W	Putative protein of unknown function
1.239	YPL277C	YPL277C	Putative protein of unknown function
1.233	YPL278C	YPL278C	Putative protein of unknown function
1.488	YPR203W	YPR203W	Putative protein of unknown function
1.316	YOL154W	ZPS1	Putative GPI-anchored protein

Down-regulated genes

LogRatio	ORF	Gene	Description
**Cell wall organization**			
-1.234	YLR110C	CCW12	Cell wall mannoprotein
-0.945	YLR390W-A	CCW14	Covalently linked cell wall glycoprotein
-1.894	YKL096W	CWP1	Cell wall mannoprotein that localizes to birth scars
-0.988	YBL043W	ECM13	Non-essential protein of unknown function
-1.247	YBL101C	ECM21	Regulation of endocytosis of plasma membrane proteins
-0.969	YMR176W	ECM5	Subunit of the Snt2C complex
-1.122	YPL221W	FLC1	Flavin adenine dinucleotide transporter
-1.412	YJL159W	HSP150	O-mannosylated heat shock protein
-1.161	YPR159W	KRE6	Type II integral membrane protein
-0.904	YDL146W	LDB17	Protein involved in the regulation of endocytosis
-1.041	YOL047C	LDS2	Protein Involved in spore wall assembly
-1.746	YGL178W	MPT5	mRNA-binding protein of the PUF family
-1.404	YGR023W	MTL1	Putative plasma membrane sensor
-2.213	YKL163W	PIR3	O-glycosylated covalently-bound cell wall protein
-1.881	YDR055W	PST1	Cell wall protein containing putative GPI-attachment site
-1.248	YBR005W	RCR1	Protein involved in cell wall chitin deposition
-0.996	YPL066W	RGL1	Regulator of Rho1p signaling, cofactor of Tus1p
-2.339	YDR077W	SED1	Major stress-induced structural GPI-cell wall glycoprotein
**Plasma membrane transactions**			
-3.408	YCL025C	AGP1	Low-affinity, broad specificity amino acid permease
-0.933	YJL170C	ASG7	Protein that regulates signaling from G protein Ste4p
-5.099	YDR046C	BAP3	Amino acid permease
-1.143	YNL192W	CHS1	Chitin synthase I
-1.029	YIR032C	DAL3	Ureidoglycolate lyase
-2.424	YDR040C	ENA1	P-type ATPase sodium pump
-2.706	YDR039C	ENA2	P-type ATPase sodium pump
-0.930	YDR373W	FRQ1	N-myristoylated calcium-binding protein
-1.059	YBR021W	FUR4	Plasma membrane localized uracil permease
-1.136	YLR343W	GAS2	1,3-beta-glucanosyltransferase
-1.014	YKR106W	GEX2	Proton:glutathione antiporter
-2.827	YDR508C	GNP1	High-affinity glutamine permease
-1.120	YGR191W	HIP1	High-affinity histidine permease
-1.694	YFL014W	HSP12	Plasma membrane organization protein
-1.729	YMR011W	HXT2	High-affinity glucose transporter
-2.171	YLR413W	INA1	Protein of unknown function
-4.585	YGR121C	MEP1	Ammonium permease
-3.648	YPR138C	MEP3	Ammonium permease of high capacity and low affinity
-1.969	YDR033W	MRH1	Protein that localizes primarily to the plasma membrane
-3.227	YGR055W	MUP1	High affinity methionine permease
-2.070	YOL084W	PHM7	Protein of unknown function
-1.519	YBR296C	PHO89	Plasma membrane Na+/Pi cotransporter
-3.345	YOR161C	PNS1	Protein of unknown function
-0.918	YLR414C	PUN1	Plasma membrane protein with a role in cell wall integrity
-1.230	YKL051W	SFK1	Membrane protein generating normal levels of PI4P
-2.002	YDR011W	SNQ2	Plasma membrane ATP-binding cassette transporter
-2.543	YDR536W	STL1	Glycerol proton symporter of the plasma membrane
-3.600	YBR069C	TAT1	Valine, leucine, isoleucine, and tyrosine transporter
-2.883	YOL020W	TAT2	High affinity tryptophan and tyrosine permease

-1.314	YGR260W	TNA1	High affinity nicotinic acid plasma membrane permease
-0.997	YBR293W	VBA2	Permease of basic amino acids in vacuolar membrane
-1.522	YLL048C	YBT1	Transporter of the ATP-binding cassette (ABC) family
-1.574	YGR281W	YOR1	Plasma membrane ATP-binding cassette transporter
-0.987	YIR039C	YPS6	Putative GPI-anchored aspartic protease
-1.070	YNL093W	YPT53	Stress-induced Rab family GTPase
-1.353	YGL255W	ZRT1	High-affinity zinc transporter of the plasma membrane

Biosynthesis of small molecules, including aminoacids

-0.955	YMR120C	ADE17	Enzyme of 'de novo' purine biosynthesis
-1.112	YBR145W	ADH5	Alcohol dehydrogenase isoenzyme V
-1.110	YFL030W	AGX1	Alanine:glyoxylate aminotransferase (AGT)
-1.864	YMR169C	ALD3	Cytoplasmic aldehyde dehydrogenase
-0.993	YDR380W	ARO10	Phenylpyruvate decarboxylase
-1.929	YCR005C	CIT2	Peroxisomal citrate synthase, involved in glyoxylate cycle
-1.308	YOR100C	CRC1	Mitochondrial inner membrane carnitine transporter
-1.625	YEL071W	DLD3	2-OH-glutarate transhydrogenase, and D-lactate dh
-0.993	YAL026C	DRS2	Trans-golgi network aminophospholipid translocase
-0.978	YNL191W	DUG3	Component of glutamine amidotransferase (GATase II)
-2.701	YCL026C-A	FRM2	Type II nitroreductase, using NADH as reductant
-1.034	YMR250W	GAD1	Glutamate decarboxylase
-1.819	YDR019C	GCV1	T subunit of the mitochondrial glycine decarboxylase
-1.591	YMR189W	GCV2	P subunit of the mitochondrial glycine decarboxylase
-1.585	YOR375C	GDH1	NADP(+)-dependent glutamate dehydrogenase
-1.231	YIL053W	GPP1	Constitutive DL-glycerol-3-phosphate phosphatase
-1.083	YER062C	GPP2	DL-glycerol-3-phosphate phosphatase
-1.007	YCL030C	HIS4	Multifunctional enzyme of histidine biosynthesis
-0.970	YIL020C	HIS6	Enzyme catalyzing fourth step in histidine pathway
-1.067	YLR099C	ICT1	Lysophosphatidic acid acyltransferase
-0.938	YOR136W	IDH2	Subunit of mitochondrial isocitrate dehydrogenase
-1.690	YML056C	IMD4	Inosine monophosphate dehydrogenase
-1.170	YIR034C	LYS1	Saccharopine dehydrogenase (NAD+, L-lysine-forming)
-1.166	YDL182W	LYS20	Homocitrate synthase functioning in DNA repair
-1.016	YFR030W	MET10	Subunit alpha of assimilatory sulfite reductase
-1.000	YNL277W	MET2	L-homoserine-O-acetyltransferase
-0.900	YBR084W	MIS1	Mitochondrial C1-tetrahydrofolate synthase
-1.428	YKR097W	PCK1	Phosphoenolpyruvate carboxykinase
-1.082	YGL037C	PNC1	Nicotinamidase converting nicotinamide to nicotinic acid
-1.155	YLR180W	SAM1	S-adenosylmethionine synthetase
-1.881	YMR095C	SNO1	Protein of unconfirmed function
-2.365	YMR096W	SNZ1	Protein involved in vitamin B6 biosynthesis
-2.376	YHR139C	SPS100	Protein required for spore wall maturation
-1.331	YJR009C	TDH2	Glyceraldehyde-3-phosphate dehydrogenase, isozyme 2
-1.774	YGR144W	THI4	Thiazole synthase
-0.974	YMR271C	URA10	Minor orotate phosphoribosyltransferase isozyme
-1.248	YNL160W	YGP1	Cell wall-related secretory glycoprotein
-1.778	YHR033W	YHR033W	Putative protein of unknown function
-1.053	YJL070C	YJL070C	Putative metallo-dependent hydrolase family protein

Response to stress

-0.946	YNL219C	ALG9	Mannosyltransferase, involved in N-linked glycosylation
-1.449	YPL250C	ATG41	Protein of unknown function
-1.517	YOR028C	CIN5	Basic leucine zipper TF of the yAP-1 family

-1.958	YGR088W	CTT1	Cytosolic catalase T
-0.982	YFL053W	DAK2	Dihydroxyacetone kinase
-2.409	YOL052C-A	DDR2	Multi-stress response protein
-1.417	YHR043C	DOG2	2-deoxyglucose-6-phosphate phosphatase
-1.388	YER035W	EDC2	RNA-binding protein that activates mRNA decapping
-1.921	YDR534C	FIT1	Mannoprotein that is incorporated into the cell wall
-1.399	YDL022W	GPD1	NAD-dependent glycerol-3-phosphate dehydrogenase
-4.548	YPL223C	GRE1	Hydrophilin essential in desiccation-rehydration process
-2.503	YOL151W	GRE2	methylbutanal reductase and NADPH-dependent methylglyoxal reductase
-1.152	YLR113W	HOG1	MAP kinase involved in osmoregulation
-1.422	YGR043C	NQM1	Transaldolase of unknown function
-1.646	YMR174C	PAI3	Cytoplasmic proteinase A (Pep4p) inhibitor
-1.258	YMR105C	PGM2	Phosphoglucomutase
-1.149	YBR092C	PHO3	Constitutively expressed acid phosphatase
-1.295	YMR175W	SIP18	Phospholipid-binding hydrophilin
-1.699	YPL130W	SPO19	Meiosis-specific prospore protein
-1.477	YBL075C	SSA3	ATPase involved in protein folding and the response to stress
-1.008	YLR006C	SSK1	Cytoplasmic phosphorelay osmosensor and regulator
-1.081	YDR074W	TPS2	Phosphatase subunit of the trehalose-6-P synthase
-1.151	YMR261C	TPS3	Regulatory subunit of trehalose-6-phosphate synthase
-1.257	YML100W	TSL1	Large subunit of trehalose 6-phosphate synthase
-1.298	YDR134C	YDR134C	Cell wall protein
-0.955	YJL144W	YJL144W	Hydrophilin essential in desiccation-rehydration process

Ribosome biogenesis

-1.081	YAL059W	ECM1	Pre-ribosomal factor involved in 60S subunit export
-0.919	YPL226W	NEW1	ATP binding cassette protein
-0.994	YPR144C	NOC4	Nucleolar protein
-0.976	YER126C	NSA2	Protein constituent of 66S pre-ribosomal particles
-1.148	YPR102C	RPL11A	Ribosomal 60S subunit protein L11A
-1.113	YKL006W	RPL14A	Ribosomal 60S subunit protein L14A
-1.104	YBL087C	RPL23A	Ribosomal 60S subunit protein L23A
-1.017	YGL031C	RPL24A	Ribosomal 60S subunit protein L24A
-1.521	YGR148C	RPL24B	Ribosomal 60S subunit protein L24B
-1.097	YLR344W	RPL26A	Ribosomal 60S subunit protein L26A
-1.039	YLR406C	RPL31B	Ribosomal 60S subunit protein L31B
-1.101	YDL191W	RPL35A	Ribosomal 60S subunit protein L35A
-0.988	YDL136W	RPL35B	Ribosomal 60S subunit protein L35B
-1.159	YLR325C	RPL38	Ribosomal 60S subunit protein L38
-1.451	YDL184C	RPL41A	Ribosomal 60S subunit protein L41A
-1.085	YPR043W	RPL43A	Ribosomal 60S subunit protein L43A
-0.929	YLR448W	RPL6B	Ribosomal 60S subunit protein L6B
-1.400	YGL076C	RPL7A	Ribosomal 60S subunit protein L7A
-0.978	YML091C	RPM2	Protein subunit of mitochondrial RNase P
-1.101	YDL081C	RPP1A	Ribosomal stalk protein P1 alpha
-1.151	YOL039W	RPP2A	Ribosomal protein P2 alpha
-1.072	YBR048W	RPS11B	Protein component of the small (40S) ribosomal subunit
-1.169	YJL191W	RPS14B	Protein component of the small (40S) ribosomal subunit
-1.358	YML024W	RPS17A	Ribosomal protein 51 (rp51) of the small (40s) subunit
-1.396	YML026C	RPS18B	Protein component of the small (40S) ribosomal subunit
-0.931	YJR145C	RPS4A	Protein component of the small (40S) ribosomal subunit
-2.372	YBL072C	RPS8A	Protein component of the small (40S) ribosomal subunit
-1.121	YER102W	RPS8B	Protein component of the small (40S) ribosomal subunit
-1.101	YBR189W	RPS9B	Protein component of the small (40S) ribosomal subunit

-1.011	YDL229W	SSB1	ATPase - ribosome-associated molecular chaperone
-0.939	YPR016C	TIF6	Constituent of 66S pre-ribosomal particles
-1.061	YJR041C	URB2	Protein required for normal metabolism of the rRNA primary transcript
-1.281	YJL109C	UTP10	Nucleolar protein
-1.078	YGR283C	YGR283C	Putative methyltransferase

RNA metabolism

-0.973	YHR081W	LRP1	Nuclear exosome-associated nucleic acid binding protein
-0.900	YOR116C	RPO31	RNA polymerase III largest subunit C160
-1.532	YHR087W	RTC3	Protein of unknown function involved in RNA metabolism
-2.122	YNR034W	SOL1	Protein with a possible role in tRNA export

Endomembrane system

-1.204	YOR152C	ATG40	Autophagy receptor with a role in endoplasmic reticulum degradation
-1.244	YLL014W	EMC6	Member of conserved ER transmembrane complex
-1.521	YMR292W	GOT1	Homodimeric protein that is packaged into COPII vesicles
-0.917	YDR492W	IZH1	Membrane protein involved in zinc ion homeostasis
-1.052	YNL297C	MON2	Protein with a role in endocytosis and vacuole integrity
-1.087	YGL010W	MPO1	Protein involved in metabolism of phytosphingosine
-1.313	YDL232W	OST4	Subunit of the oligosaccharyltransferase complex of the ER lumen
-1.153	YLR073C	RFU1	Protein that inhibits Doa4p deubiquitinating activity
-0.916	YDR233C	RTN1	Reticulon protein
-1.046	YLL016W	SDC25	Non-essential Ras guanine nucleotide exchange factor (GEF)

Mitochondrial metabolism

-1.121	Q0070	AI5_ALPHA	Endonuclease I-SceIV
-1.177	YGR110W	CLD1	Mitochondrial cardiolipin-specific phospholipase
-1.176	YMR256C	COX7	Subunit VII of cytochrome c oxidase (Complex IV)
-1.293	YJR048W	CYC1	Cytochrome c, isoform 1
-1.845	YGR052W	FMP48	Putative protein of unknown function
-1.497	YBR004C	GPI18	Functional ortholog of human PIG-V
-1.328	YJR091C	JSN1	Member of the Puf family of RNA-binding proteins
-1.026	YKL150W	MCR1	Mitochondrial NADH-cytochrome b5 reductase
-1.087	YHR162W	MPC2	Highly conserved subunit of the mitochondrial pyruvate carrier (MPC)
-1.644	YGR243W	MPC3	Highly conserved subunit of the mitochondrial pyruvate carrier (MPC)
-4.944	YJL116C	NCA3	Protein involved in mitochondrion organization
-1.337	YHR001W-A	QCR10	Subunit of the ubiqunol-cytochrome c oxidoreductase complex
-1.229	YGR183C	QCR9	Subunit 9 of ubiquinol cytochrome-c reductase (Complex III)
-1.359	YLR251W	SYM1	Protein required for ethanol metabolism
-1.297	YJL045W	YJL045W	Minor succinate dehydrogenase isozyme

Genome maintenance

-1.019	YGL251C	HFM1	Meiosis specific DNA helicase
-1.371	YDR013W	PSF1	Subunit of the GINS complex (Sld5p, Psf1p, Psf2p, Psf3p)
-1.092	YLR329W	REC102	Protein involved in early stages of meiotic recombination
-0.985	YKR101W	SIR1	Protein involved in silencing at mating-type loci HML and HMR

Protein modification

-0.920	YJR036C	HUL4	Protein with similarity to hect domain E3 ubiquitin-protein ligases
-0.977	YGR123C	PPT1	Protein serine/threonine phosphatase

Transcription factors

-1.332	YBR239C	ERT1	Transcriptional regulator
-1.438	YJL110C	GZF3	GATA zinc finger protein

Pexophagy

-1.306	YJL185C	ATG36	Pex3p interacting protein, required for pexophagy

Invasive growth

-1.598	YMR316W	DIA1	Protein of unknown function

Zinc ion homeostasis

-1.066	YOL101C	IZH4	Membrane protein involved in zinc ion homeostasis

Nuclear pore complex

-1.119	YLL023C	POM33	Transmembrane nucleoporin

Unknown function

-1.284	YHR126C	ANS1	Putative GPI protein
-2.012	YLR267W	BOP2	Protein of unknown function
-1.501	YGR146C	ECL1	Protein of unknown function
-1.642	YDR070C	FMP16	Protein of unknown function
-1.772	YHR049W	FSH1	Putative serine hydrolase
-1.129	YPL280W	HSP32	Possible chaperone and cysteine protease
-1.353	YOR391C	HSP33	Possible chaperone and cysteine protease
-1.011	YHR151C	MTC6	Protein of unknown function
-2.457	YJL108C	PRM10	Pheromone-regulated protein
-1.077	YMR034C	RCH1	Putative transporter
-1.490	YGR213C	RTA1	Protein involved in 7-aminocholesterol resistance
-2.367	YMR118C	SHH3	Putative mitochondrial inner membrane protein of unknown function
-1.104	YMR322C	SNO4	Possible chaperone and cysteine protease
-1.617	YBR150C	TBS1	Protein of unknown function
-2.786	YBR032W	YBR032W	Putative protein of unknown function
-0.902	YBR063C	YBR063C	Putative protein of unknown function
-0.976	YDR034W-B	YDR034W-B	Predicted tail-anchored plasma membrane protein
-0.965	YEL067C	YEL067C	Putative protein of unknown function
-0.976	YER039C-A	YER039C-A	Putative protein of unknown function
-2.690	YER053C-A	YER053C-A	Protein of unknown function
-1.145	YGL193C	YGL193C	Haploid-specific gene repressed by a1-alpha2
-1.763	YGL258W-A	YGL258W-A	Putative protein of unknown function
-1.295	YGR066C	YGR066C	Putative protein of unknown function
-1.535	YGR201C	YGR201C	Putative protein of unknown function
-2.313	YHR022C	YHR022C	Putative protein of unknown function
-1.135	YIL029C	YIL029C	Putative protein of unknown function
-1.687	YJL107C	YJL107C	Putative protein of unknown function
-1.014	YJR039W	YJR039W	Putative protein of unknown function
-1.655	YJR115W	YJR115W	Putative protein of unknown function
-0.983	YKL066W	YKL066W	Dubious open reading frame
-1.752	YKL071W	YKL071W	Putative protein of unknown function
-1.422	YKR011C	YKR011C	Protein of unknown function
-1.052	YLR030W	YLR030W	Putative protein of unknown function
-1.110	YLR031W	YLR031W	Putative protein of unknown function
-0.956	YLR046C	YLR046C	Putative membrane protein
-1.171	YLR108C	YLR108C	Protein of unknown function
-1.124	YML002W	YML002W	Putative protein of unknown function
-1.466	YMR102C	YMR102C	Protein of unknown function
-1.287	YMR181C	YMR181C	Protein of unknown function
-3.009	YMR244W	YMR244W	Putative protein of unknown function
-1.273	YNL195C	YNL195C	Protein of unknown function

-1.809	YNR014W	YNR014W	Putative protein of unknown function
-0.910	YOR309C	YOR309C	Dubious open reading frame
-0.938	YOR385W	YOR385W	Putative protein of unknown function
-1.214	YPL035C	YPL035C	Dubious open reading frame
-1.161	YPL088W	YPL088W	Putative aryl alcohol dehydrogenase
-1.513	YGL259W	YPS5	Protein with similarity to GPI-anchored aspartic proteases

Table S2 Transcriptional factors (TFs) most strongly involved in the regulation of genes selected in our transcriptome analysis. The list was generated with the help of the Yeastract+ web tool and database [20] (http://www.yeastract.com). Each TF name is accompanied by the description of function according to SGD and the list of genes that are regulated by that TF according to Yeastract+. See the Results section for a more detailed description of these parameters and the filtering strategy.

Up-regulated genes

TF	Rank	Description	Regulated genes
Adr1p	11.1	Carbon source-responsive zinc-finger transcription factor; required for transcription of the glucose-repressed gene ADH2, of peroxisomal protein genes, and of genes required for ethanol, glycerol, and fatty acid utilization	*ADH2, ALD4, ALD5, ASP3-1, ASP3-3, ATP11, BAT2, BRF1, BTN2, COX17, COX23, CTA1, DCG1, DDI2, DIC1, ECI1, ENT1, ERG6, FAA2, FMC1, FOX2, FSF1, GAT2, GDH2, HPT1, HXT17, HXT6, IBD2, ICL2, IDP3, LAP3, LPX1, MOD5, MRPL31, MRPL44, OAC1, PGU1, PHM6, POX1, Q0010, QDR1, QDR3, RRN11, SFG1, SPS19, YEL076C, YEL076C-A, YER138W-A, YFL067W, YHR214W-A, YIG1, YKR075C, YMR193C-A, YNL122C*
Mga1p	9.4	Protein similar to heat shock transcription factor; multicopy suppressor of pseudohyphal growth defects of ammonium permease mutants	*ADH2, AIM34, ALD4, ASP3-1, ASP3-2, ASP3-3, ASP3-4, ATP11, BAT2, BTN2, CDC21, COX17, COX23, CSM3, DCG1, DDI3, ERG6, FIR1, GAT2, HIS7, HXT17, IPL1, LAS17, LEU2, MAM33, MCD1, MIF2, MRPL31, MTH1, OAC1, OYE3, PCL1, PHM6, POL32, POX1, RKM1, SEN34, SEO1, SFG1, SFH5, SGO1, SHO1, SPS19, STP4, TOS2, TUB4, YBL107W-A, YER138W-A, YFL068W, YHP1, YKR075C, YOR389W, YPL278C, YUH1*
Fzf1p	6.8	Transcription factor involved in sulfite metabolism; sole identified regulatory target is SSU1; overexpression suppresses sulfite-sensitivity of many unrelated mutants due to hyperactivation of SSU1	*BAT2, CDA1, CDC21, CTK1, DDI2, DDI3, DSN1, DUR3, FRE5, HIS7, PCL1, POX1, SET5, SGO1, TOS4, YJR011C, YPT10, YSP3*
Gzf3p	5.1	GATA zinc finger protein; negatively regulates nitrogen catabolic gene expression by competing with Gat1p for GATA site binding; function requires a repressive carbon source; dimerizes with Dal80p and binds to Tor1p	*AIM34, BTN2, CDC21, DAL1, DAL4, DAL5, DAL7, DAL80, DCG1, DUR3, FIR1, FMC1, HPA3, MIG3, MRPL44, PCL1, PHM6, POL32, PUT1, RRN11, SFG1, SFH5, SGO1, STP4, THI80, TOS4, YHP1, YIG1, YKR075C, YOR389W, YPL278C*
Dal81p	4.8	Positive regulator of genes in multiple nitrogen degradation pathways	*ALD4, ALD5, BAT2, BTN2, DAL1, DAL4, DAL5, DAL7, ERF2, GAT2, GDH2, LEU2, MTH1, NRK1, OAC1, OPT2, PCL1, PHM6, PUT1, QDR3, SFG1, SFH5, YGK3, YLR257W*
Cat8p	4.6	Zinc cluster transcriptional activator; necessary for derepression of a variety of genes under non-fermentative growth conditions, active after diauxic shift, binds carbon source responsive elements	*AAC3, ADH2, ALD4, CTA1, DIC1, FOX2, GDH2, LEU2, LPX1, MF(ALPHA)2, OAC1, POX1, PRO3, RRN11, TOS4, YSP3*
Dal80p	3.6	Negative regulator of genes in multiple nitrogen degradation pathways; expression is regulated by nitrogen levels and by Gln3p; member of the GATA-binding family, forms homodimers and heterodimers with Gzf3p	*ASP3-1, BAT2, DAL1, DAL4, DAL5, DAL7, DAL80, DCG1, DUR3, HPA3, IFM1, MRP13, PHM6, PUT1, TOS4*
Tod6p	3.1	PAC motif binding protein involved in rRNA and ribosome biogenesis; subunit of the RPD3L histone deacetylase complex; Myb-like HTH transcription factor	*ADH2, BTN2, CDC21, DCG1, DSN1, MIG3, PCL1, STP4, TOS4, YPT10*
Sut2p	2.1	Zn2Cys6 family transcription factor; positively regulates sterol uptake under anaerobic conditions with SUT1; represses filamentation-inducing genes during non-starvation conditions; positively regulates mating	*ALD4, ASP3-1, BAT2, BUD8, CDA1, CDC21, DSN1, ERF2, GAT2, PCL1, SUT2, TOS4, YPT10*
Wtm2p	2.0	Transcriptional modulator; involved in regulation of meiosis, silencing, and expression of RNR genes; involved in response to replication stress	*BAT2, CDC21, DSN1, FRE5, GAT2, IPI1, PCL1, RAD27, SGO1, STP4, TOS4, YLR257W*
Rpi1p	1.5	Transcription factor, allelic differences between S288C and Sigma1278b; mediates fermentation stress tolerance by modulating cell wall integrity	*BAT2, CDC21, DBP8, DSN1, FRE5, GAT2, PCL1, TOS4, YDL211C, YLR257W, ZDS2*

Down-regulated genes

TF	Rank	Description	Regulated genes
Hot1p	126.0	Transcription factor for glycerol biosynthetic genes; required for the transient induction of glycerol biosynthetic genes GPD1 and GPP2 in response to high osmolarity; targets Hog1p to osmostress responsive promoters	*ALD3, CTT1, ENA1, FIT1, FLC1, FMP48, GPD1, GPP1, GPP2, GRE1, GRE2, HSP12, MPC3, NQM1, PAI3, PHM7, PNC1, PNS1, RTC3, SED1, SFK1, SIP18, SPS100, STL1, THI4, TPS2, TSL1, URA10, YGP1, YGR066C, YJL107C, YNL195C*
Crz1p	40.0	Transcription factor, activates transcription of stress response genes; nuclear localization is positively regulated by calcineurin-mediated dephosphorylation	*AGP1, ALD3, ARO10, ASG7, ATG40, CCW12, CCW22, CHS1, CIT2, CLD1, COX7, CTT1, CWP1, CYC1, DAL3, DDR2, DIA1, DRS2, ECM13, ECM21, EDC2, ENA1, ENA2, ERT1, FLC1, FMP16, FMP48, FSH1, FUR4, GAD1, GCV2, GDH1, GNP1, GPD1, GPI18, GPP1, GPP2, HSP12, HSP150, HXT2, ICT1, IDH2, INA1, IZH4, KRE6, LYS20, MEP1, MET2, MPC2, MPC3, MPO1, MRH1, MUP1, NQM1, NSA2, PCK1, PGM2, PHM7, PHO3, PHO89, PIR3, PNC1, PNS1, PUN1, QCR9, RCH1, RCR1, RGL1, ROQ1, RPL11A, RPL24A, RPL24B, RPL38, RPP1A, RTA1, RTC3, SDC25, SED1, SIP18, SNO1, SNQ2, SNZ1, SPS100, STL1, SYM1, THI4, TPS2, TPS3, TSL1, YDR034W-B, YGP1, YGR201C, YGR283C, YHR033W, YLR046C, YLR108C, YMR102C, YNL195C, YNR014W, YOR309C, YOR385W, YPT53*
Gis1p	33.5	Histone demethylase and transcription factor; regulates genes during nutrient limitation	*AGX1, ALD3, ANS1, BAP3, BOP2, CIN5, CIT2, COX7, CRC1, CTT1, CWP1, CYC1, DAL3, DDR2, DIA1, ECM13, ECM21, ERT1, FMP16, FMP48, GAD1, GDH1, GEX2, GPD1, GPP1, GPP2, GRE1, GRE2, HSP12, HSP32, HSP33, HXT2, IZH4, LYS1, MEP1, MPO1, MUP1, NQM1, PAI3, PCK1, PGM2, PHM7, PHO89, PIR3, PNS1, POM33, QCR10, ROQ1, RPP2A, RTC3, SDC25, SHH3, SIP18, SNO4, SPS100, SSA3, STL1, SYM1, TAT1, TAT2, TPS2, URA10, YDR034W-B, YGP1, YGR066C, YGR201C, YHR022C, YJL045W, YLR031W, YMR181C, YNL195C, YNR014W, YPS6*
Rlm1p	29.4	MADS-box transcription factor; component of the protein kinase C-mediated MAP kinase pathway involved in the maintenance of cell integrity	*ALD3, ARO10, BOP2, CCW12, CCW14, CHS1, CTT1, CWP1, DDR2, ECL1, ECM13, FLC1, FMP16, FMP48, FRQ1, GAD1, GPD1, GPP1, GPP2, GRE1, GRE2, HOG1, HSP12, HSP150, HUL4, ICT1, INA1, KRE6, MCR1, MPC3, MTL1, NCA3, NOC4, NQM1, NSA2, PAI3, PCK1, PGM2, PHM7, PHO3, PIR3, PNC1, PNS1, PRM10, PST1, PUN1, RCR1, RPL24A, RTA1, RTC3, SED1, SHH3, SIP18, SNO4, SPS100, STL1, SYM1, TAT2, TPS2, TSL1, UTP10, YDR034W-B, YGL258W-A, YGP1, YGR066C, YHR033W, YJL107C, YJR115W, YLR031W, YNL195C, YNR014W, YOR309C, YPL088W, YPS5, YPS6, YPT53, ZRT1*
Ifh1p	20.7	Coactivator, regulates transcription of ribosomal protein (RP) genes	*ADH5, DLD3, ECL1, FRM2, GAS2, GDH1, HIS4, HIS6, HSP12, IMD4, IZH4, LYS20, NCA3, PIR3, RPL11A, RPL14A, RPL23A, RPL24A, RPL24B, RPL26A, RPL31B, RPL35A, RPL35B, RPL38, RPL41A, RPL43A, RPL6B, RPL7A, RPP1A, RPP2A, RPS11B, RPS14B, RPS17A, RPS18B, RPS4A, RPS8A, RPS8B, RPS9B, RTA1, SNZ1, YDR034W-B, YKR011C, YLR030W, YLR108C*
Met4p	18.7	Leucine-zipper transcriptional activator; responsible for regulation of sulfur amino acid pathway	*ADH5, ALD3, ARO10, ATG41, CCW12, CCW14, CCW22, CTT1, CWP1, DDR2, DLD3, DUG3, ECM13, FMP16, GAD1, GCV1, GCV2, GPD1, GPP1, GPP2, GRE2, HSP12, HSP33, HXT2, LRP1, MET10, MET2, MRH1, MUP1, NEW1, NQM1, PHO3, PIR3, PST1, PUN1, RCH1, RPL24A, RPL24B, RPL26A, RPL35A, RPL35B, RPL7A, RPS11B, RPS17A, RPS4A, RPS9B, RTC3, SAM1, SIR1, SNO4, SNZ1, SSA3, STL1, TDH2, TPS2, URA10, UTP10, YGP1, YHR022C, YHR033W, YMR181C, YOR309C, ZRT1*

Mig1p	18.2	Transcription factor involved in glucose repression; regulates filamentous growth along with Mig2p in response to glucose depletion	*ALD3, ARO10, CIT2, CLD1, CTT1, CYC1, DDR2, DOG2, ECL1, ECM5, EDC2, ENA1, FMP16, FMP48, GAD1, GCV2, GPD1, GRE2, HSP12, HSP33, HXT2, ICT1, INA1, IZH1, IZH4, KRE6, MPC3, MPO1, MRH1, NQM1, PAI3, PHM7, PHO3, PHO89, PIR3, PNC1, PNS1, POM33, RCH1, RPM2, RTC3, SDC25, SED1, SNO4, TAT1, TPS2, TSL1, YDR034W-B, YGP1, YHR022C, YJL107C, YKL066W, YMR102C, YMR181C, YNL195C, YNR014W, YPS5*
Mga2p	17.6	ER membrane protein involved in regulation of OLE1 transcription; inactive ER form dimerizes and one subunit is then activated by ubiquitin/proteasome-dependent processing followed by nuclear targeting	*ADE17, AGP1, AGX1, ALD3, BOP2, CCW22, CHS1, CIT2, CLD1, CTT1, DDR2, ECL1, ECM1, EDC2, FLC1, FMP48, GAD1, GCV2, GOT1, GPD1, GPI18, GPP2, GRE1, GRE2, HSP12, HSP150, ICT1, IMD4, INA1, IZH1, IZH4, LDS2, MCR1, MEP1, MET10, MET2, MPC3, MPT5, MUP1, NEW1, PGM2, PHM7, PHO3, PNC1, PPT1, PRM10, PUN1, RCH1, RFU1, ROQ1, RPL14A, RPL23A, RPL24A, RPL24B, RPL26A, RPL31B, RPL35B, RPL38, RPL43A, RPL6B, RPL7A, RPP1A, RPP2A, RPS14B, RPS17A, RPS18B, RPS4A, RPS8A, RPS8B, RPS9B, RTA1, RTC3, SED1, SNZ1, SOL1, SPS100, SSB1, SYM1, THI4, TNA1, TPS2, TSL1, YDR034W-B, YGP1, YKR011C, YNL195C, YOR309C, YPL088W, YPT53, ZRT1*
Stp2p	16.1	Transcription factor; activated by proteolytic processing in response to signals from the SPS sensor system for external amino acids; activates transcription of amino acid permease genes	*AGP1, ARO10, ASG7, ATG40, BAP3, BOP2, CLD1, COX7, CRC1, CWP1, DAL3, DIA1, ECL1, EDC2, FMP16, FRM2, GAD1, GCV1, GDH1, GNP1, ICT1, INA1, LDS2, MCR1, MET10, MPC2, MUP1, NQM1, PAI3, PHO89, POM33, PPT1, PUN1, RCH1, RCR1, RGL1, ROQ1, RPM2, RTA1, RTC3, SDC25, SED1, SFK1, SOL1, SYM1, YBR032W, YGP1, YJL045W, YLR031W, YPL088W, YPS6, ZRT1*
Stp1p	14.8	Transcription factor; undergoes proteolytic processing by SPS sensor component Ssy5p in response to extracellular amino acids; activates transcription of amino acid permease genes and may have a role in tRNA processing	*AGP1, ARO10, ASG7, ATG40, ATG41, BAP3, BOP2, CLD1, COX7, CRC1, DAL3, DIA1, ECL1, EDC2, FIT1, FMP16, FRM2, GAD1, GCV1, GDH1, GNP1, GPD1, HIS4, ICT1, IMD4, INA1, KRE6, LDB17, LDS2, LYS1, LYS20, MCR1, MET10, MET2, MPC2, MTC6, MUP1, NQM1, PAI3, PHO89, PNC1, POM33, PPT1, PSF1, PUN1, RCH1, RCR1, ROQ1, RPM2, RTA1, RTC3, SDC25, SED1, SFK1, SOL1, SYM1, TAT1, TAT2, TSL1, YBR032W, YGP1, YGR283C, YJL045W, YLR031W, YML002W, YMR102C, YOR385W, YPL088W, YPS6, ZRT1*
Nrg1p	14.0	Transcriptional repressor; mediates glucose repression and negatively regulates a variety of processes including filamentous growth and alkaline pH response	*AGP1, ASG7, BOP2, CCW22, COX7, CRC1, CTT1, CWP1, CYC1, DAL3, DIA1, DLD3, DOG2, ECM13, EDC2, ENA1, FMP16, FMP48, GNP1, HIS6, HSP12, HXT2, ICT1, IDH2, INA1, MCR1, MEP3, PCK1, PGM2, PHM7, PHO89, PNS1, PRM10, RCR1, RPM2, SED1, SIR1, SNQ2, STL1, TAT1, TBS1, YHR022C, YHR033W, YJL107C, YMR102C, ZRT1*
Gzf3p	13.8	GATA zinc finger protein; negatively regulates nitrogen catabolic gene expression by competing with Gat1p for GATA site binding; function requires a repressive carbon source; dimerizes with Dal80p and binds to Tor1p	*AGP1, ANS1, ARO10, CCW12, CIN5, CIT2, CWP1, CYC1, DAL3, ECL1, ECM13, ECM5, ENA1, ERT1, FMP48, FSH1, GCV1, GCV2, GEX2, GPP1, GPP2, GZF3, HIS4, HSP12, ICT1, IZH4, LRP1, LYS1, MEP1, MEP3, PHO3, RCH1, RCR1, RTC3, SED1, SIP18, SNO1, SNZ1, SPS100, STL1, YBT1, YGP1, YJL107C, YLR108C, YNR014W, ZRT1*
Sko1p	13.6	Basic leucine zipper transcription factor of the ATF/CREB family; cytosolic and nuclear protein involved in osmotic and oxidative stress responses	*AGP1, AGX1, ALD3, ATG41, CCW12, CIN5, CTT1, CWP1, DAL3, DDR2, ECL1, ENA1, FIT1, FLC1, FMP48, FSH1, GAS2, GCV1, GCV2, GNP1, GPD1, GPP1, GPP2, GRE1, GRE2, GZF3, HIS4, HOG1, HSP12, HSP32, HSP33, HXT2, ICT1, IZH4, LYS1, LYS20, MCR1, MET10, MPC3, NQM1, PAI3, PHM7, PHO3, PIR3, PNC1, PNS1, POM33, PRM10, QCR9, RCH1, RPL11A, RPL24B, RPL35B, RPS17A, RPS4A, RTC3, SED1, SFK1, SIP18, SNO4, SNQ2, SOL1, SPS100, STL1, TDH2, THI4, TPS2, TSL1, URB2, UTP10, YER053C-A, YHR022C, YHR033W, YJL107C, YJR115W, YLR108C, YPS5*

Gat4p	13.3	Protein containing GATA family zinc finger motifs; involved in spore wall assembly	*AGX1, ALD3, ARO10, BOP2, CTT1, CWP1, CYC1, DDR2, ECM13, FMP48, FRM2, GAD1, GPP1, GPP2, GRE2, HSP12, HSP32, HSP33, HXT2, ICT1, IZH4, MUP1, PAI3, PCK1, PHM7, PHO3, PHO89, PIR3, PNS1, ROQ1, RTA1, RTC3, SHH3, SNO4, SPS100, SSA3, YDR034W-B, YGP1, YGR066C, YGR201C, YLR108C, YNR014W*
Nrg2p	13.0	Transcriptional repressor; mediates glucose repression and negatively regulates filamentous growth	*ASG7, COX7, CRC1, CTT1, CYC1, DAL3, DOG2, ENA1, FMP16, IDH2, MCR1, PGM2, PHO89, PNS1, RPM2, TAT1, YHR033W, ZRT1*
Hap1p	13.0	Zinc finger transcription factor; involved in the complex regulation of gene expression in response to levels of heme and oxygen	*ATG40, CCW12, CCW22, CIN5, CLD1, COX7, CTT1, CWP1, CYC1, DDR2, ECM13, EDC2, ENA1, ERT1, FMP48, GNP1, GPP2, GRE1, HOG1, HSP12, HXT2, IZH1, IZH4, LYS20, MCR1, MPC3, NCA3, PAI3, PCK1, PHM7, PHO3, PHO89, PIR3, QCR10, QCR9, RPL23A, RPL38, RPM2, RPS4A, RTN1, SAM1, SED1, SFK1, SNQ2, STL1, TBS1, TSL1, YER053C-A, YGL193C, YGP1, YHR022C, YJL107C, YJR115W, YLR108C, YMR102C, YOR1*
Rgm1p	10.9	Putative zinc finger transcription factor; overproduction impairs cell growth and induces expression of genes involved in monosaccharide catabolism and aldehyde metabolism; regulates expression of subtelomeric genes	*ALD3, CHS1, COX7, CTT1, CWP1, CYC1, DDR2, DIA1, FIT1, FMP16, GAD1, GAS2, GPD1, GPP2, GRE2, HSP12, ICT1, MCR1, MET10, PCK1, PGM2, PNC1, POM33, PST1, QCR10, QCR9, RCH1, RGL1, RPL24B, RPP2A, RPS11B, RPS18B, RTC3, SAM1, SED1, SFK1, SNQ2, SNZ1, STL1, SYM1, TIF6, TPS2, TSL1, YGP1, YKL071W, YLR030W, YML002W, YMR181C, YPL088W*
Sfl1p	10.5	Transcriptional repressor and activator; involved in repression of flocculation-related genes, and activation of stress responsive genes; has direct role in INO1 transcriptional memory	*ADE17, ADH5, ASG7, BAP3, CCW12, CCW14, CWP1, DDR2, DIA1, DRS2, ECL1, EDC2, FMP48, FRM2, GAS2, GCV2, GRE2, HIS4, HSP12, HSP32, HSP33, ICT1, IZH4, LDS2, LYS1, PHM7, PHO89, PIR3, PNS1, PRM10, PUN1, RCR1, ROQ1, RTA1, SIP18, SNO1, SNO4, SNQ2, SNZ1, TAT2, YDR034W-B, YER039C-A, YER053C-A, YGL258W-A, YGR066C, YJL107C, YJR115W, YLR108C, YMR244W, YOR385W, YPS5*
Tbs1p	6.2	Putative transcription factor of unknown function	*COX7, CWP1, CYC1, FMP48, HSP12, LDS2, LYS20, PHM7, QCR9, RCH1, YGP1, YLR108C, YNL195C*
Rof1p	6.1	Putative transcription factor containing a WOPR domain	*ASG7, CHS1, DIA1, EDC2, HXT2, IZH1, MRH1, NQM1, PUN1, RCR1, ROQ1, RPL43A, RPL6B, RTA1, STL1, YGL193C, YGP1, YHR033W, YJL045W, YLR108C, YMR244W, YOR385W, YPS5, YPS6*
Tog1p	5.9	Transcriptional activator of oleate genes; regulates genes involved in fatty acid utilization	*CHS1, CLD1, GAD1, GAS2, GPD1, ICT1, IZH4, LDS2, PCK1, PGM2, PST1, QCR10, RCH1, ROQ1, SFK1, SNQ2, SYM1, TPS2, TSL1, YGP1, YMR181C*
Wtm2p	5.1	Transcriptional modulator; involved in regulation of meiosis, silencing, and expression of RNR genes; involved in response to replication stress	*CHS1, GAD1, GPD1, GPI18, ICT1, IZH4, PGM2, PST1, PUN1, RCH1, SFK1, SYM1, TPS2, TSL1, YGP1, YLR108C, YMR181C*
Rpi1p	4.8	Transcription factor, allelic differences between S288C and Sigma1278b; mediates fermentation stress tolerance by modulating cell wall integrity	*CHS1, GAD1, GZF3, IZH4, KRE6, PGM2, PST1, PUN1, RCH1, RTA1, SFK1, SOL1, SYM1, TPS2, TSL1, YMR181C, YOR385W*
Com2p	4.3	Transcription factor; *COM2* transcription is regulated by Haa1p, Sok2p and Zap1p transcriptional activators; C. albicans homolog (MNL1) plays a role in adaptation to stress	*CHS1, GAD1, GPD1, IZH4, PGM2, PST1, RCH1, RGL1, RTC3, SFK1, SNQ2, SYM1, TPS2, TSL1, YGP1, YMR181C*
Sdd4p	3.5	Putative transcription factor, induced in response to the DNA-damaging agent MMS	*CCW14, GAD1, GPI18, MEP3, PST1, PUN1, RCH1, SFK1, VBA2, YGP1, YMR181C, YPL088W*
